# Chinese herbal medicine for constipation: *zheng*-based associations among herbs, formulae, proprietary medicines, and herb–drug interactions

**DOI:** 10.1186/s13020-016-0099-4

**Published:** 2016-06-23

**Authors:** Linda L. D. Zhong, Guang Zheng, Li Da Ge, Cheng Yuan Lin, Tao Huang, Ling Zhao, Cheng Lu, Ai Ping Lu, Zhao Xiang Bian

**Affiliations:** School of Chinese Medicine, Hong Kong Baptist University, 7 Baptist University Road, Kowloon Tong, Hong Kong China; Hong Kong Chinese Medicine Study Centre, Hong Kong Baptist University, Hong Kong SAR, China; Department of Surgery, The First People’s Hospital of Xiaoshan District, Hangzhou, China; Institute of Basic Research in Clinical Medicine, China Academy of Traditional Chinese Medicine, Beijing, China; School of Information Science and Engineering, Lanzhou University, Lanzhou, China

## Abstract

**Background:**

As current symptomatic treatments of constipation are still unsatisfactory, an increasing number of patients seek help from Chinese medicine (CM), particularly Chinese herbal medicine (CHM). This study aimed to review the most frequently used CHM herbs and formulae, proprietary CHMs, and herb–drug interactions for functional constipation using *zheng* (syndrome)-based differentiation, and to determine the current practice of *zheng*-based CHM treatments for functional constipation.

**Methods:**

We developed a search strategy to include all the related clinical studies of CHM for constipation and set inclusion and exclusion criteria as studies on subjects with constipation of all ages and both sexes, using objective measures from laboratory or imaging techniques. The interventions included single herbs, CM classical formulae, CM new formulae, and Chinese herb-derived products and combination products. The clinical study types included were quasi- or randomized controlled trials, observational clinical studies, case series or case reports, and other types of appropriate research methods. The data concerning study design, sample size, mode of recruitment, sampling and diagnostic procedure, inclusion and exclusion criteria, and participants’ characteristics (including age, sex, and duration of constipation). CM patterns, CM treatment principles, treatment regimen, and CM treatment outcomes were recorded.

**Results:**

A total of 29,832 relevant records were found, of which 8541 were duplicate records and 20,639 were excluded for reasons of irrelevance. The full text of 965 articles was retrieved for detailed assessment, following which 480 articles were excluded for various reasons. From the included articles, we retrieved 190 different CM *zheng* diagnoses from 485 individual studies. The most common *zheng* was *dual deficiency of qi and blood (N* = *48)*, which was diagnosed in 948 out of 15,740 subjects. The most frequently used classical formula was *Ma*-*Zi*-*Ren*-*Wan* (MZRW) (N = 75) and the most frequently used proprietary CHM was *Run*-*Chang*-*Wan (N* = *87)*. The most frequently used combined medication was *Da Huang* with sodium bicarbonate tablets (frequency across all studies, n = 23), followed by *Fan Xie Ye* with lactulose oral solution (n = 8), *Ma*-*Ren*-*Ruan*-*Jiao*-*Nang* with lactulose oral solution (n = 6) and *Liu*-*Wei*-*An*-*Xiao*-*Jiao*-*Nang* (n = 6) with mosapride citrate tablets.

**Conclusion:**

This study examined the use of CHM for constipation and summarized the herbs, formulae, proprietary medicines, and herb–drug interactions application. These data indicated there were limited information about herb-drug interactions and adverse effects of CHM and further randomized controlled trials with strict design are necessary.

**Electronic supplementary material:**

The online version of this article (doi:10.1186/s13020-016-0099-4) contains supplementary material, which is available to authorized users.

## Background

Constipation is a common functional bowel disorder that affects many people; 14.7 % of the United States population [1], and 15.6 % of the adult population in Hong Kong [2] experienced this problem in a large sampled cross-sectional survey published in 2014. Treatments for constipation usually include fiber supplements, osmotic and stimulant laxatives, stool softeners, and sometimes enemas for refractory constipation [3]. As current symptomatic treatments produce unsatisfactory responses [4], many patients seek help from Chinese medicine (CM), particularly Chinese herbal medicine (CHM).

Many CM interventions have been used to treat constipation. A recent review [5] listed the current clinical research findings from CM interventions for functional constipation. However, there have been no analysis of the benefits of individual interventions (or individual types of interventions) or of the qualities of individual study designs. Our research team conducted a systematic review of CHM for functional constipation [6] and showed that CHM or CHM combination therapy was more effective than some single conventional medicines [6]. However, these findings did not accurately reflect all clinical practice, as most clinical research on constipation has involved observational studies or case series, and clinical practice has mostly been limited to personal experiences and based on CM theory and *zheng* (syndrome) differentiation [7–9].

We aimed to investigate CHM applications for constipation based on *zheng* differentiation, especially the use of single herbs, CM formulae, proprietary CHMs, and herb–drug interactions. Therefore, we systematically reviewed all the available data from current databases, including clinical trials, clinical observational studies, case series, case reports, and case control studies. Because we examined large data sets from both conventional Western and CM literature, we used a data slicing algorithm for text mining [10].

This study aimed to review the most frequently used CHM herbs and formulae, proprietary CHMs, and herb–drug interactions for functional constipation using *zheng*-based differentiation, and to determine the current practice of *zheng*-based CHM treatments for functional constipation.

## Methods

### Literature search

The review was performed in accordance with the Preferred Reporting Items for Systematic Reviews and Meta-Analyses (PRISMA) guidelines. We used the following databases to search the conventional medicine literature: PubMed, Ovid, Evidence-Based Medicine Reviews (EBMR), and Embase. The following databases were used to search the traditional CM literature: SinoMed, Chinese National Knowledge Infrastructure (CNKI), Chinese biomedical literature (CBM) CD, and China Journals Full-text Database. From the electronic database records and bibliographic references, we identified relevant primary sources and secondary sources (such as textbooks, review articles, and meta-analyses) as follows. We selected all EBM reviews, including Cochrane DSR, ACP Journal Club, DARE, CCTR, CMR, HTA, and NHSEED from inception to April 2014; EMBASE from 1980 to April 2014; EMBASE Classic from 1947 to 1979; PubMed from inception to April 2014; Ovid MEDLINE(R) from 1950 to April 2014; Ovid OLDMEDLINE(R) from 1948 to 1965; SinoMed from 1978 to April 2014; China Journals Full-text Database from 1994 to April 2014 and CBM disc from 1979 to April 2014. The search strategy was (1) (constipation) OR (chronic constipation) OR (functional constipation); (2) (herb*) OR (herbal medicine) OR (traditional Chinese medicine) OR (Chinese medicine) OR (Complementary medicine) OR (Naturopathy); (3) (case*) OR (clinical observation*) OR (clinical trial) OR (clinical study); (1) AND (2) AND (3) (*was used for truncation).

### Study selection

We included interventions using single herbs, CM classical formulae, CM new formulae, and Chinese herb-derived products and combination products. The clinical study types included were quasi- or randomized controlled trials, observational clinical studies, case series or case reports, and other types of appropriate research methods. We included studies on subjects with constipation of all ages and both sexes, studies using objective measures from laboratory or imaging techniques, and studies using measurement from nursing staff, patients, or other informants.

### Data extraction

Two authors (LLDZ and GZ) independently searched the databases and selected relevant publications. If the two authors disagreed about a study’s eligibility, they would check the study against the selection criteria, discuss its eligibility, and make a further decision (ZXB). One author (LLDZ) extracted the data and the other (GZ) checked the extracted data. For each study, the following information was extracted: study design, sample size, mode of recruitment, sampling and diagnostic procedure, inclusion and exclusion criteria, and participants’ characteristics (including age, sex, and duration of constipation). CM patterns, CM treatment principles, treatment regimen, and CM treatment outcomes were recorded.

### Quality assessment

The methodological quality of relevant studies was assessed using the Jadad scale (Additional file 1; [11]). The Jadad scale evaluates a study in terms of the description of randomization, blinding, and dropouts. The scale ranges from 1 to 5; randomized controlled trials with a score between 3 and 5 are regarded as better quality trials. Points were awarded if the study was described as randomized (1 point), had an appropriate randomization method (1 point), was described as double-blind (1 point), used an appropriate blinding method (1 point), and had a description of withdrawals and dropouts (1 point) [11].

### Identification of herb-*zheng* associations

Classification based on keyword co-occurrence was conducted on the 18,272 items of constipation literature downloaded from SinoMed [12]. We also applied a dictionary-based data slicing algorithm constructed on the principle of keyword co-occurrence. We filtered the downloaded data using CM associated keywords, such as “Chinese herbal medicine,” “Chinese patent medicine,” and “CM syndrome/*zheng*,” which were obtained from textbooks and the Internet. The keyword co-occurrence classification was a good supplement to the literature search, as it provided insights into the quantitative relationship between the individual herbs and formulae used to treat constipation. We used a wheel-shaped network to indicate the association between different types of single herbs and their z*heng* indications [10]. The wheel-shaped network was a visualized graph that showed the frequencies and correlations among the same categories [10].

### Translation of terminology

All Chinese-to-English translations were deduced primarily from the *World Health Organization (WHO) Evidence*-*Based Complementary and Alternative Medicine International Standard Terminologies on Traditional Medicine in the Western Pacific Region* [13].

## Results

**Fig. 1:**
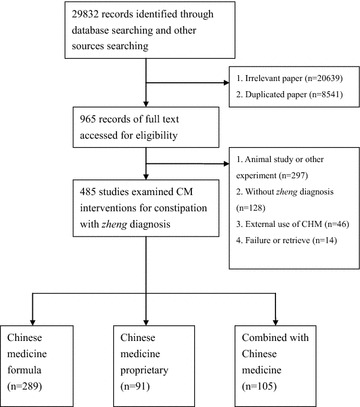
Flowchart of literature selection logistics

We accessed 29,832 records that fit the search criteria, of which 8541 were duplicate records and 20,639 were excluded for reasons of irrelevance. The full text of 965 articles was retrieved for detailed assessment; 480 of these were excluded for various reasons (Fig. 1). Of the 485 studies that fulfilled the inclusion and exclusion criteria, 289 were on CM formulae, 91 were on proprietary CM, and 105 were on a combination of CHM and conventional treatment. The sample size of the 485 studies ranged from 35 to 250. Among all the studies, 289 (59.6 %) were case series or reports, 125 (25.8 %) were controlled trials, and 71 (14.6 %) were randomized controlled trials. For the randomized controlled trials, the mean Jadad scores were 2.06 and their average quality was quite low (Additional file 1).

### CM *zheng* category and treatment criteria

From the included articles, we retrieved 190 different CM *zheng* diagnoses from 485 individual studies. The most common pattern was *dual deficiency of qi and blood (Qi Xue Liang Xu)*, which was diagnosed in 948 of the 15,740 subjects (frequency = 48, percentage among the top 10 diagnosis = 16.8 %); this was followed by *dual deficiency of qi and yin (Qi Yin Liang Xu)* (subjects = 795, frequency = 45, percentage among the top 10 diagnosis = 15.7 %), *excessive heat and qi stagnation (Qi Zhi Shi Re)* (subjects = 726, frequency = 41, percentage among the top 10 diagnosis = 14.3 %), *yang deficiency of spleen and kidney (Pi Shen Yang Xu)* (subjects = 636, frequency = 32, percentage among the top 10 diagnosis = 11.2 %), *deficiency of qi and blood (Qi Xue Liang Xu)* (subjects = 595, frequency = 26, percentage among the top 10 diagnosis = 9.1 %), *cold*–*heat complex (Han Re Cuo Za)* (subjects = 513, frequency = 21, percentage among the top 10 diagnosis = 7.3 %), *liver depression and spleen deficiency (Gan Yu Pi Xu)* (subjects = 495, frequency = 20, percentage among the top 10 diagnosis = 7.0 %), *deficiency*–*excess complex (Xu Shi Jia Za)* (subjects = 483, frequency = 20, percentage among the top 10 diagnosis = 7.0 %), *dualyin deficiency of liver and kidney (Gan Shen Yin Xu)* (subjects = 410, frequency = 17, percentage among the top 10 diagnosis = 5.9 %) and *intestinal dryness and yin deficiency (Yin Xu Chang Zao)* (subjects = 223, frequency = 16, percentage among the top 10 diagnosis = 5.6 %). Subjects diagnosed with the top 10 CM *zheng* accounted for 37 % of the 15,740 subjects (Table 1). Table 1 lists the therapeutic principles for the CM *zheng*.

**Table 1:** Top ten most commonly used CM *zheng* for constipation

CM *zheng*	Therapeutic principle	Number of subjects diagnosed by the diagnosis	Number of frequency among all the studies	Percentage among the total *zheng* (190)/top 10 *zheng* diagnosis (%)
*Dual deficiency of qi and blood qi*-*xue*-*liang*-*xu*	Tonify *qi* and replenish *blood*	948	48	16.8/6.2
*Dual deficiency of qi and yin qi*-*yin*-*liang*-*xu*	Tonify *qi* and replenish *yin*	795	45	15.7/5.9
*Excessive heat and qi stagnation qi*-*zhi*-*shi*-*re*	Soothe the *liver* and regulate *qi*	726	41	14.3/5.3
*Yang deficiency of spleen and kidney pi*-*shen*-*yang*-*xu*	Warm the *kidney* and fortify the spleen	636	32	11.2/4.2
*Deficiency of qi and blood pi*-*xue*-*kui*-*xu*	Tonify *qi* and engender *blood*	595	26	9.1/3.4
*Cold*-*heat complex han*-*re*-*cuo*-*za*	Treat *cold* with *heat* and *heat* with *cold*	513	21	7.3/2.7
*Liver depression and spleen deficiency gan*-*yu*-*pi*-*xu*	Soothe the *liver* and fortify the *spleen*	495	20	7.0/2.6
*Deficiency*-*excess complex xu*-*shi*-*jia*-*za*	Treat *deficiency* by tonification and excess by purgation	483	20	7.0/2.6
*Dual* *yin deficiency of liver and kidney gan*-*shen*-*yin*-*xu*	Enrich the *kidney* and nourish the *liver yin*	410	17	5.9/2.2
*Intestinal dryness and yin deficiency yin*-*xu*-*chang*-*zao*	Replenish *yin* and moisten dryness	223	16	5.6/2.1

### CM herbs and their relationships

**Fig. 2:**
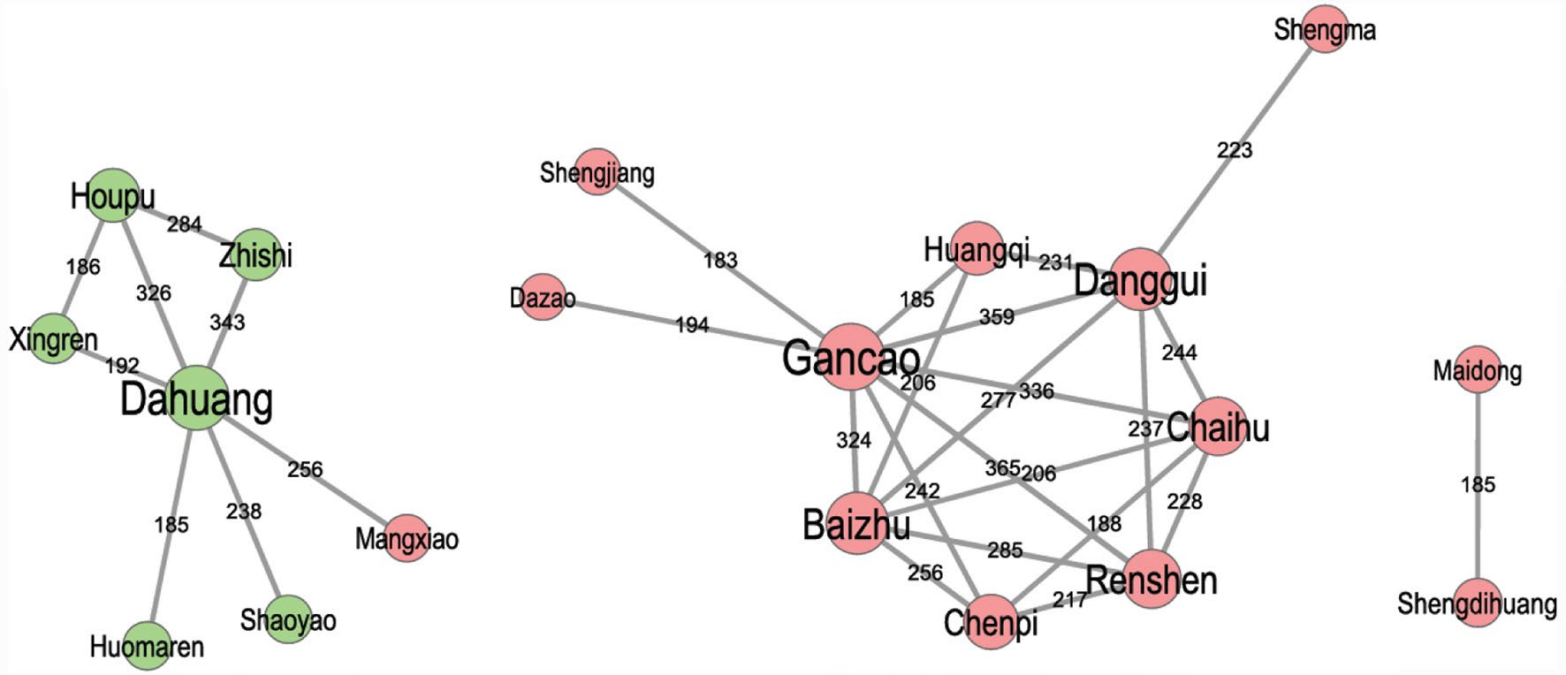
Network relationship of Chinese herbal medicine in the treatment of constipation. *Red* and *green* nodes represent different single herbal medicines. The *edges* represent co-occurrence frequency in clinical studies. The *edge label numbers* represent the number of clinical studies demonstrating a connection between two single herbal medicines

We identified 296 herbs from 485 clinical studies and analyzed their relationships using the wheel-shaped network (Fig. 2). In this figure, red and green nodes represent different single herbal medicines. The edges represent co-occurrence frequency in clinical studies. The edge label numbers represent the number of clinical studies demonstrating a connection between two single herbal medicines. There was a high concentration of one classical Chinese herbal formula, *Ma*-*Zi*-*Ren*-*Wan*, whose composition of six herbs occupied 42.5 % (1754/4127) of the total frequency of usage. Based on this, the green nodes represent CHMs in the formula *Ma*-*Zi*-*Ren*-*Wan* and the red nodes represent other CHMs used in clinical prescriptions [10]. Node size was calculated with the formula

$$Node \, size = LOG \, \left( {node\_frequency} \right) + Degree \, (node)$$

where *node_frequency* is the literature record number of the associated keyword calculated in text mining, *LOG* is the logarithm calculation with a base set to 10, and *Degree (node)* is the number of connections/edges each node has with other nodes. The edge line width was calculated through *LOG (edge_co*-*occurrent)* where *edge_co*-*occurrent* was the number of node/keyword pairs that co-occurred in the associated literature.

Table 2 lists the top 10 most frequently used herbs and their actions. The most frequently used herbs were further categorized and analyzed to determine their combinations in classical formulae. The most frequently used classical formulae were *Ma*-*Zi*-*Ren*-*Wan* [14] and *Zeng*-*Ye*-*Tang* [15].

**Table 2:** Action and indication of the most ten frequently used herbs

Chinese name in pinyin	Latin name	Frequency of usage	Action	Indication
*Da Huang*	Radix *et Rhizoma Rhei*	428	Drains *heat* and purges accumulations; clears *heat*, transforms *dampness* and promotes urination; drains *heat* from the *blood*; invigorates the *blood* and dispels blood stasis	Intestinal *heat excess* with high fever, profuse sweating, thirst, constipation, abdominal distention and pain, delirium, a yellow tongue coat and a full pulse; blood stasis with amenorrhea, fixed abdominal masses or fixed pain
*Huo Ma Ren*	*Semen Cannabis*	247	Nourishes, moistens and lubricates the intestines; nourishes the *yin;* clears *heat* and promotes healing of sores; moistens dryness and benefits the hair	Constipation in the elderly; constipation after a warm febrile disease; postpartum constipation; constipation due to blood and *yin* *deficiency*; sores and ulcerations (auxiliary-internal and topical); promotes hair growth and treats dry hair
*Fan Xie Ye*	*Folium Sennae*	232	Drains downward and guides out stagnation; eliminates excess *heat* and drains *summer heat*	*Heat* in the stomach and intestines with constipation and abdominal fullness; *summer heat*
*Bai Zhu*	Rhizoma *Atractylodis Macrocephalae*	198	Tonifies the *spleen* and augments *qi*; dries *dampness* and promotes water metabolism; stabilizes the exterior and stops sweating	*Spleen* and *stomach deficiency* with diarrhea, fatigue; *spleen damp* or accumulation of fluids affecting the digestion; edema and reduced urination due to *spleen deficiency*; *qi* *deficiency* with spontaneous sweating
*Dang*- *Gui*	Radix *Angelicae Sinensis*	132	Tonifies the *blood* and regulates the menses; invigorates and harmonizes the *blood* and disperses cold; moistens the intestines and unblocks the bowels	Blood deficiency with pale, ashen complexion, lusterless nails, tinnitus; *blood deficiency* associated irregular menstruation, amenorrhea and dysmenorrhea; dry Intestines due to blood deficiency
*Huang Qi*	Radix *Astragali*	126	Tonifies *qi* and *blood*; strengthens the *spleen* and raises the *yang qi* of the *spleen* and *stomach*; tonifies *wei qi*, stabilizes the exterior and tonifies the *lungs*	Postpartum fever due to *qi* and *blood deficiency*; *spleen* *qi* *deficiency* with anorexia, fatigue and diarrhea; excessive sweating with *qi*, *yang* or *yin* *deficiency*; *dampness* in the head
*Gan Cao*	Radix *Glycyrrhizae*	112	Tonifies the *spleen* and augments *qi*; moistens the *lungs*, resolves phlegm and stops cough; moderates spasms and alleviates pain; clears *heat* and relieves *fire* toxicity	Spleen *qi* *deficiency* with shortness of breath, lassitude and loose stools; *qi* and *blood* *deficiency* with an irregular pulse and/or palpitations; productive or non-productive cough; raw for toxic *heat* with sore throat or carbuncles and sores
*Sheng Di*	Radix *Rehmanniae*	99	Clears *heat* and cools the *blood*; nourishes *yin*, generates fluids, increases saliva and treats wasting and thirsting; cools heart fire	*Ying*-stage *heat* with high fever, thirst and a scarlet tongue; hemorrhage due to *blood heat*; throat pain due to *yin* *deficiency*
*Bai Shao*	Radix *Paeoniae Alba*	94	Nourishes the *blood* and regulates menstruation; astringes *yin* and adjusts the *ying* and *wei*; calms *liver* *yang* and *liver* *wind* and alleviates pain	*Liver blood deficiency* with menstrual dysfunction, vaginal discharge and uterine bleeding; anemia; breast distention and pre-menstrual syndrome
*Lu Hui*	Aloe	93	Purges, drains *fire* and guides out accumulations; clears *heat* and cools the *liver*; kills parasites and strengthens the *stomach*	*Heat* accumulation with constipation, dizziness, red eyes, and irritability; chronic constipation; heat in the liver channel or liver fire with epigastric discomfort, dizziness, headache, irritability, tinnitus, constipation and fever

### CM *zheng*-based Chinese herbal formulae

Among the 289 studies on Chinese herbal formulae, the most frequently used formulae based on *zheng* diagnosis were *Ma*-*Zi*-*Ren*-*Wan* and its modifications (frequency among all the studies, n = 75, percentage among the top 10 formulae, 33.07 %). This was followed by *Bu*-*Zhong*-*Yi*-*Qi*-*Tang* (n = 56, 22.58 %), *Ji*-*Chuan*-*Jian* (n = 51, 20.56 %), *Zeng*-*Ye*-*Tang* (n = 40, 13.84 %), and *Ba*-*Zhen*-*Tang* (n = 26, 10.48 %). The five most frequently used CM *zheng*-based Chinese herbal formulae and their indications are summarized in Table 3.

**Table 3:** Summary of top five most frequently used Chinese herbal formulae based on *zheng* diagnosis

Chinese name in pinyin	Composition in pinyin	CM *zheng*	Number of frequency among all the studies	Actions in Chinese medicine
*Ma*-*Zi*-*Ren*-*Wan*	*Huo Ma Ren* *Xing Ren* *Bai Shao* *Zhi Shi* *Hou Pu* *Da Huang*	Excessive *qi* and *heat*	75	Invigorates *Blood* Dispels *Blood* Stasis Moves *qi* Lubricates the intestines Moves the bowels Purges *heat* Alleviates pain
*Bu*-*Zhong*-*Yi*-*Qi*-*Tang*	*Huang Qi* *Ren Shen* *Bai Zhu* *Zhi Gan Cao* *Dang Gui* *Chen Pi* *Shen Ma* *Chai Hu*	*Qi deficiency* of *spleen* and *stomach*/*Sunken middle qi*	56	Tonifies *middle* *jiao qi* Benefits *qi* Regulates *qi* Raises *sunken* *yang* Lifts prolapsed organs
*Ji*-*Chuan*-*Jian*	*Dang Gui* *Niu Xi* *Rou Cong Rong* *Ze Xie* *Shen Ma* *Zhi Qiao*	*Yang deficiency* of *spleen* and *kidney*	51	Warms up the *kidney* Replenishes vital essence Lubricates the intestines Induces defecation
*Zeng*-*Ye*-*Tang*	*Xuan Shen* *Mai Dong* *Sheng Di*	*Fluid*-*humor deficiency*	40	Generates Fluids Moistens dryness Unblocks the bowels Nourishes *yin* Clears *heat*
*Ba*-*Zhen*-*Tang*	*Ren Shen* *Bai Zhu* *Fu Ling* *Zhi Gan Cao* *Shu Di* *Bai Shao* *Chuan Xiong* *Dang Gui*	*Dual deficiency* of *qi* and *blood*	26	Nourishes *qi* Benefits *blood*

### Proprietary CHMs

After the classical herbal decoctions, the next most frequently used clinical treatments for constipation were proprietary CHMs, because of their standard quality control and more convenient administration. We analyzed the most commonly used proprietary CHMs and their dosage (Table 4). The manufacturers of the proprietary CHMs are also shown, to indicate the quality and composition of the medicines. The most commonly used proprietary CHM was *Run*-*Chang*-*Wan* (frequency, n = 87), followed by *Ma*-*Ren*-*Ruan*-*Jiao*-*Nang* (n = 62), *Ma*-*Ren*-*Run*-*Chang*-*Wan* (n = 52), *Liu*-*Wei*-*An*-*Xiao*-*Jiao*-*Nang* (n = 50), *Fu*-*Fang*-*Lu*-*Hui*-*Jiao*-*Nang* (n = 35), *Si*-*Ni*-*San* (n = 32), *Liu*-*Wei*-*Neng*-*Xiao*-*Jiao*-*Nang* (n = 27), and *Bu*-*Zhong*-*Yi*-*Qi*-*Wan* (n = 17).

**Table 4:** Summary of top ten most frequently used Chinese herbal medicine proprietary

Chinese name in pinyin (manufacturer)	Composition in pinyin	Oral dosage	Number of frequency among all the studies	Indication
*Run*-*Chang*-*Wan* (Shang Dong Hua Yang Pharmaceutical Co. Ltd.)	*Tao Ren*	4 pills t. i. d. for oral administration	87	Constipation with CM *zheng* of *excessive q*i and *heat*, and constipation for elderly people and postpartum women
	*Da Hua*			
	*Qiang Huo*			
	*Dang Gui*			
	*Huo Ma Ren*			
*Ma*-*Ren*-*Ruan*-*Jiao*-*Nang* (Actavis (Foshan) Pharmaceutical Co., Ltd.)	*Huo Ma Ren*	2 capsules t. i. d. for oral administration	62	Constipation with CM *zheng* of *excessive qi* and *heat*
	*Xing Ren*			
	*Bai Shao*			
	*Zhi Shi*			
	*Hou Pu*			
	*Da Huang*			
*Ma*-*Ren*-*Run*-*Chang*-*Wan* (Bei Jing Tong Ren Tang Co., Ltd.)	*Huo Ma Ren*	1–2 big honey pills b. i. d. for oral administration	52	Constipation with CM *zheng* of *heat* in *stomach* and *intestines*
	*Xing Ren*			
	*Da Hua*			
	*Bai Shao*			
	*Mu Xiang*			
	*Chen Pi*			
*Liu*-*Wei*-*An*-*Xiao*-*Jiao*-*Nang* (Gui Zhou Xin Bang Pharmaceutical Co., Ltd.)	*Tu Mu Xiang*	3–6 pills b. i. d. for oral administration	50	Constipation due to indigestion and bloating; stomachache; dyspepsia
	*Da Huang*			
	*Shan Nai*			
	*Han Shui Shi*			
	*Ke Zi*			
	*Jian Hua*			
*Fu*-*Fang*-*Lu*-*Hui*-*Jiao*-*Nang* (He Bei Wan Bang & Folon Pharmaceutical Co., Ltd.)	*Lu Hui*	1–2 capsules b. i. d. for oral administration	35	Constipation with CM *zheng* of intense *fire* in *heart* and *liver*
	*Qing Dai*			
	*Zhu Sha*			
	*Hu Po*			
*Ma*-*Ren*-*Zi*-*Pi*-*Wan* (Bao Tou Chinese Medicine Pharmaceutical Co., Ltd.)	*Huo Ma Ren*	1 big honey pills b. i. d. for oral administration	34	Constipation with CM *zheng* of dryness and heat in large intestine
	*Xing Ren*			
	*Bai Shao*			
	*Zhi Shi*			
	*Hou Pu*			
	*Da Huang*			
	*Yu Li Ren*			
	*Dang Gui*			
*Si*-*Ni*-*San* (Fu Zhou Neptunus Futao Pharmaceutical Co., Ltd.)	*Chai Hu*	6–9 g granules b. i. d. for oral administration	32	Constipation with CM *zheng* of disharmony between *spleen* and *liver*
	*Zhi Shi*			
	*Bai Shao*			
	*Zhi Gan Cao*			
*Liu*-*Wei*-*Neng*-*Xiao*-*Jiao*-*Nang* (Yang Zong Pharmaceutical Co., Ltd.)	*Da Hua*	2 capsules b. i. d. for oral administration	27	Constipation due to indigestion; obesity; hyperlipidemia
	*Ke Zi*			
	*Gan Jiang*			
	*Zang Mu Xiang*			
	*Jian Hua*			
	*Hai Shui Shi*			
*Bu*-*Zhong*-*Yi*-*Qi*-*Wan* (Bei Jing Tong Ren Tang Co., Ltd.)	*Huang Qi*	1–2 big honey pills b. i. d. for oral administration	17	Constipation with CM *zheng* of *qi deficiency* of *spleen* and *stomach*/*Sunken middle qi*
	*Dang Shen*			
	*Bai Zhu*			
	*Dang Gui*			
	*Shen Ma*			
	*Chai Hu*			
	*Chen Pi*			
	*Zhi Gan Cao*			

*t.i.d.* means ter in die, three times a day; *b.i.d.* means bis in die, twice a day

### CM combined with Western medicine and adverse effects

Few clinical studies reported the combined use of CM and Western medicine for constipation. Table 5 summarized the herb–drug interactions identified. Although most studies did not mention the adverse effects associated with combined administration of herbs and drugs, or proprietary CHMs and drugs, there were 43 reported trials of the integrative use of single herbs or proprietary CHMs. Among these studies, the most frequent adjunctive use was *Da Huang* (*Radix et Rhizoma Rhei*) with *sodium bicarbonate tablets* (n = 23), followed by *Fan Xie Ye* (*Folium Sennae*) with *lactulose oral solution* (n = 8), *Ma*-*Ren*-*Ruan*-*Jiao*-*Nang* with *lactulose oral solution* (n = 6) and *Liu*-*Wei*-*An*-*Xiao*-*Jiao*-*Nang withmosapride citrate tablets* (n = 6).

**Table 5:** Summary of combination of CHM and Western medicine and the reported adverse effects

	Combined used Western medicine	Frequency of reported in all the trials (n > 5)	Adverse effect (N = , percentage, %)
Single herb			
*Da Huang*	Sodium bicarbonate tablets	23	Symptoms of the gastrointestinal tract (N = 4, 17.3 %)
			Insomnia (N = 1, 4.3 %)
			Skin rash (N = 1, 4.3 %)
			Headache (N = 1, 4.3 %)
*Fan Xie Ye*	Lactulose oral solution	8	Symptoms of the gastrointestinal tract (N = 2, 25 %)
			Dizzy and anorexia (N = 1, 12.5 %)
CHM Proprietary			
*Ma*-*Ren*-*Ruan*-*Jiao*-*Nang*	Lactulose oral solution	6	Symptoms of the gastrointestinal tract (N = 2, 33.3 %)
			Headache (N = 1, 16.7 %)
*Liu*-*Wei*-*An*-*Xiao*-*Jiao*-*Nang*	Mosapride citrate tablets	6	Symptoms of the gastrointestinal tract (N = 3, 50 %)

## Discussion

To our knowledge, this is the first study to examine the use of single herbs, classical CM formulae, proprietary CHMs, and the combined use of CM and Western medicine (and its adverse effects) for constipation. The classification of single herbs and CM formulae was generally based on the CM diagnostic *zheng* system. As we pointed out in our commentary paper, *zheng* diagnosis is a critical stage of CM treatment; it is the basis of CM’s effectiveness and the main feature that distinguishes it from Western medicine [16]. In this review, we focused on *zheng* diagnosis, in accord with current clinical CM practice. Although we identified 190 different CM *zheng* diagnoses, only 57.9 % (281/485) of studies used the diagnosis system of *zheng* differentiation. The most commonly diagnosed *zheng* for constipation was *dual deficiency of qi and blood* (16.8 %) followed by *dual deficiency of qi and yin* (15.7 %) and *excessive heat and qi stagnation* (14.3 %). As Table 1 showed, among the top 10 CM *zheng*, five belonged to *deficiencyzheng* and the others could be categorized as a combination of *deficiency* and *sufficiency* or *sufficiency* syndrome. These results are consistent with those of our previous study [16] and with other research on syndrome distribution among constipation patients [8, 17], which indicated that almost half of patients, especially older individuals and postpartum women, had *deficiency* syndromes [18, 19].

Of the 10 most frequently used herbs, the top three were traditional purgatives; the other herbs were *tonifying* and *replenishing* medicines, except *LuHui* (*Aloe vera*) [20, 21]. It is interesting that the top five CM *zheng* were *deficiencyzheng*; this seemed inconsistent with the fact that the five most frequently used single herbs were purgatives. This was mainly because only 50.26 % of studies used CM *zheng* differentiation, and purgatives are mostly used for general constipation in the absence of any CM *zheng* diagnosis. Table 3 shows that the most commonly used Chinese herbal formula was *Ma*-*Zi*-*Ren*-*Wan*, which suggested that it formed the basis of the most commonly prescribed formulae for constipation according to both CM syndrome differentiation and Western medicine diagnosis.

Many studies of Chinese herbal formulae or proprietary CHM did not provide standard or complete criteria for syndrome diagnosis. The lack of detail and consistency in diagnosis makes these studies difficult to replicate and their findings difficult to compare with other results [22]. Most studies provided only the main composition of the formulae without any indication of dosage or quality control standards (Table 4). Although proprietary CHMs are rapidly gaining attention in the West as sources of new drugs, dietary supplements, and functional foods, the lack of consistent manufacturing processes, quality standards, scientific evidence, and validation of efficacy and safety impede worldwide acceptance of CHM [23].

Currently, herb–drug interactions are of growing concern as a clinical safety issue to clinicians and researchers [24, 25]. Proprietary CHMs are prescribed by Western medical doctors who may not fully understand the indications and actions of these medicines [26]. Although herbal medicines are natural, they are not always safe [27]. Table 5 showed the concomitant use and the adverse effects of herb–drug combinations or proprietary CHM–drug combinations based on the limited literature. These limited data showed that the incidence of adverse effects was not as low as we expected, ranging from 4.3 to 50 %, although most adverse effects were symptoms of the gastrointestinal tract.

A limitation of this study was that the data were drawn from clinical studies that used several different types of design: case reports, cohort studies, and quasi- or randomized controlled trials. The quality of these studies varied and therefore it is difficult to compare them quantitatively. In addition, most studies did not provide sufficient detail regarding inclusion criteria of diseases and syndromes, quality control procedures for single herbs or proprietary CHMs, or withdrawal rates and reasons. Therefore, the analysis of the data derived from this study is limited and should be treated with caution.

## Conclusion

This review examined the use of CHM for constipation and summarized the most frequently used Chinese single herbs, the 10 most frequently used CHM formulae and proprietary CHMs, and the combined use of CHM and Western medicine treatments and their reported adverse effects.

## Additional file

10.1186/s13020-016-0099-4 RCT Jadad scores.

## Abbreviations

CHM: Chinese herbal medicine
CM: Chinese medicine
MZRW: *Ma*-*Zi*-*Ren*-*Wan*
PRISMA: Preferred Reporting Items for Systematic Reviews and Meta-Analyses
EBMR: Evidence-Based Medicine Reviews
CNKI: Chinese National Knowledge Infrastructure
CBM: Chinese biomedical literature
