# Supplementary material for: Chinese herbal medicine for constipation: zheng-based associations among herbs, formulae, proprietary medicines, and herb–drug interactions
Source: Chin Med. 2016 Jun 23;11:28. doi: 10.1186/s13020-016-0099-4 (PMC4919884; doi:10.1186/s13020-016-0099-4)
Supplement: Supplementary file 1 — 10.1186/s13020-016-0099-4 RCT Jadad scores. [file 13020_2016_99_MOESM1_ESM.pdf]

RCT Jadad scores

Jadad developed a five-point questionnaire (Jadad Scores) to assess the methodological quality of clinical studies [10]. The Jadad Scale comprises of three points, **randomization**, **blinding**, and **withdrawals** and dropouts. If the method of randomization or blinding is appropriate, one point is given. If the method of randomization or blinding is inappropriate, one point is deducted.

Jadad Scores (5-points)

1. *Was the study described as randomized?*
2. *Was the study described as double blind?*
3. *Was there a description of withdrawals and dropouts?*

One additional point is given for each question

1. *The method of randomization was described in the paper, and that method was appropriate.*
2. The method of blinding was described, and it was appropriate.

One additional point is deducted for each question

1. *The method of randomization was described, but was inappropriate.*
2. *The method of blinding was described, but was inappropriate.*

Number	Author	Year	Jadad Scale
1	Jiang M, Xiong N, et al	2005	1
2	Huo L, Zhang J, et al.	2006	1
3	Jiang Y, Wang L.	2008	3
4	Zhang L, Li N.	2006	1
5	Kong L.	2008	1
6	Zhan C, Wang F.	2005	1
7	Yan J, Liu Y.	2005	2
8	Huang C.	2007	1
9	Yu Y.	2007	2
10	Jin Z, Yang B, et al.	2008	2
11	Sun G, Li J, et al.	2008	1
12	Qiu B, Tang X, et al.	2008	2
13	Song S.	2010	2
14	Zheng H.	2010	2
15	Shi Z, Xu W, et al.	2009	2
16	Lin Z, Li S.	2007	1
17	Tang T, Qin J.	2009	2
18	Li F, Chen Q.	2008	1
19	Fan D.	2007	2
20	Wang H, Jin H.	2004	1
21	Guan R, Zhao J.	2005	2
22	Wu W.	2005	2
23	Sun Y, Cui Z.	2008	1
24	Xie Y, Tang X, et al.	2007	1
25	Liu Z.	2007	2
26	Qu Q.	2013	3
27	Liu X.	2004	1
28	Xie S, Feng J.	2014	3
29	Zhan C, Chen J	2011	1
30	Shi C, He Y, et al.	2009	2
31	Gan A, Zhang F.	2008	1
32	Yang T.	2008	3
33	Fan D, Qin X.	2009	2
34	Li F, Shen J.	2004	1
35	Wu RM.	2009	2
36	Cai HQ.	2004	3
37	Chen Z, Zeng E, et al.	2004	3
38	Li S, Song C.	2005	2
39	Yan L, Cao Y.	2007	1

40	Zhu Q.	2008	2
41	Sun H, Qiao Y, et al.	2008	1
42	Xiong GH.	2012	3
43	Yan X, Guo M.	2013	2
44	Wang W.	2006	1
45	Liang C, Wu X.	2008	3
46	Wang X, Zhu R.	2007	2
47	Kang Y.	2014	3
48	Wang Q.	2014	3
49	Xin H, Zhang J.	2009	3
50	Meng L.	2009	2
51	Guo S.	2013	2
52	Guo S, Xu J.	2009	2
53	Jiang X, Zhang Q, et al.	2011	2
54	Li Y, Wang J.	2008	2
55	Chen Y.	2009	3
56	Huang M.	2013	2
57	Zheng X, Xu Z.	2014	3
58	Zhang H.	2014	3
59	Jian F, Li G.	2010	2
60	Yin K.	2013	3
61	Shi C, Zhou J.	2008	3
62	Zang X, Liu R.	2010	3
63	Liu D.	2011	2
64	Guo M, Wang A.	2013	3
65	Zhao Y.	2013	3
66	Dong H, Gao Y.	2009	3
67	Ma X.	2009	1
68	Sun H.	2011	3
69	Sun J.	2011	3
70	Li J.	2009	3
71	Tan Y.	2014	3
